# Maternal Malic Acid May Ameliorate Oxidative Stress and Inflammation in Sows through Modulating Gut Microbiota and Host Metabolic Profiles during Late Pregnancy

**DOI:** 10.3390/antiox13020253

**Published:** 2024-02-19

**Authors:** Meixia Chen, Ying Zhao, Shuang Li, Zhuo Chang, Hui Liu, Dongyan Zhang, Sixin Wang, Xin Zhang, Jing Wang

**Affiliations:** 1 Institute of Animal Husbandry and Veterinary Medicine, Beijing Academy of Agriculture and Forestry Sciences, Beijing 100097, China; liuhui@baafs.net.cn (H.L.); zhangdongyan@baafs.net.cn (D.Z.); wangsixin@baafs.net.cn (S.W.); 2 Precision Livestock and Nutrition Unit, TERRA Teaching and Research Centre, Gembloux Agro-Bio Tech, University of Liège, 5030 Gembloux, Belgium; yingzhaocaas@163.com; 3 College of Animal Science and Technology, Anhui Agricultural University, Hefei 230036, China; lishuang@ahau.edu.cn; 4 Beijing General Station of Animal Husbandry, Beijing 100107, China; changzhuobeijing@163.com; 5 State Key Laboratory of Animal Nutrition and Feeding, College of Animal Science and Technology, China Agricultural University, Beijing 100193, China; zhangx0904@cau.edu.cn

**Keywords:** L-malic acid, late pregnancy, sow, gut microbiota, metabolic

## Abstract

Sows suffer oxidative stress and inflammation induced by metabolic burden during late pregnancy, which negatively regulates reproductive and lactating performances. We previously found that L-malic acid (MA) alleviated oxidative stress and inflammation and improved reproductive performances in sows. However, the mechanism underlying the MA’s positive effects remains unexplored. Here, twenty Large White × Landrace sows with similar parity were randomly divided into two groups and fed with a basal diet or a diet supplemented with 2% L-malic acid complex from day 85 of gestation to delivery. The gut microbiome, fecal short-chain fatty acids, and untargeted serum metabolome were determined. Results showed that Firmicutes, Bacteroidota, and Spirochaetota were the top abundant phyla identified in late pregnancy for sows. Maternal MA supplementation modulated the composition but not the richness and diversity of gut microbiota during late pregnancy. Correlation analysis between gut microbiota and antioxidant capacity (or inflammation indicators) revealed that *unclassified_f_Ruminococcaceae*, *unclassified_f_Lachnospiraceae*, *UCG-002*, *norank_f_norank_o_RF3*, and *Lactobacillus* might play a role in anti-oxidation, and *Lachnospiraceae_XPB1014_group*, *Lachnospiraceae_NK4A136_group*, *UCG-002*, *unclassified_f_Ruminococcaceae*, *Candidatus_Soleaferrea*, *norank_f_UCG-010*, *norank_f_norank_o_RF39*, and *unclassified_f_Lachnospiraceae* might be involved in the anti-inflammatory effect. The improved antioxidant and inflammation status induced by MA might be independent of short chain fatty acid changes. In addition, untargeted metabolomics analysis exhibited different metabolic landscapes of sows in the MA group from in the control group and revealed the contribution of modified amino acid and lipid metabolism to the improved antioxidant capacity and inflammation status. Notably, correlation results of gut microbiota and serum metabolites, as well as serum metabolites and antioxidant capacity (or inflammation indicators), demonstrated that differential metabolism was highly related to the fecal microorganisms and antioxidant or inflammation indicators. Collectively, these data demonstrated that a maternal dietary supply of MA can ameliorate oxidative stress and inflammation in sows through modulating gut microbiota and host metabolic profiles during late pregnancy.

## 1. Introduction

Reproductive performance is highly associated with production efficiency in pig farming and well-being in humans. Pregnant mothers face consistent stress during the whole production process, especially the late pregnancy period [1]. Take sows, for example, they suffer from aggressive oxidative and inflammation stress mainly derived from dramatic fetal development-induced metabolic burden and maternal metabolic alteration during late pregnancy, which deteriorates fetal intrauterine growth and reproductive outcomes, such as farrowing duration, litter size, live litter size and litter weight gain [2,3,4]. This stress is not fully uncovered until lactation ends, which will further decrease sows’ feed intake and aggravate negative energy balance, body weight and milk production loss during lactation [5,6]. Consequently, the growth performances of offspring from the oxidative-stress-disturbed sows are adversely affected [7]. Therefore, proposing effective strategies to mitigate oxidative and inflammation stress during late pregnancy in sows is of vital significance.

Recent research has enlightened us as to the association between perinatal gut microflora and maternal oxidative stress and inflammation status [8,9]. Discernible shifts occur in the gut microbiota of the mothers during the perinatal period in the sow and humans [10,11]. Highly reproductive mothers had lower microbial richness during late pregnancy and higher microbial diversity during the early stages after parturition than their less reproductive counterparts [8]. Specifically, maternal *Bacteroides_f_Bacteroidaceae* positively correlated but *Phascolarctobacterium and Streptococcus* negatively correlated with the litter performance and the antioxidant capacity of sows [12]. A functional link analysis between gut bacteria and oxidative indicators and stillbirth rate identified that *Lachnospiraceae_UCG-001*, *Marvinbryantia*, and *Ruminococcaceae_UCG-004* were negatively correlated with antioxidant capacity, but positively correlated with the stillbirth rate in sows [13]. Furthermore, perinatal maternal gut microbiota alterations paralleled the anti-inflammation status change in sows [8]. Changes in gut microbiota responded to the host metabolism and affected maternal metabolic health status through metabolizing relevant nutrients and then affected fetal development through breast transmission [14,15]. In the late stages of pregnancy, there was a disruption in the maternal gut microbiota, characterized by a significant increase in *Proteobacteria* and *Actinobacteria* [10]. Additionally, pregnant and lactating sows exhibited low-level inflammation and metabolic disturbances [11]. Therefore, intestinal flora changes and their related metabolic changes may be the underlying mechanism of the induced antioxidant and anti-inflammatory effects and phenotypes.

Nutritional manipulation has proven to be a potent strategy to alleviate the oxidative and inflammation stress of sows [16]. Common antioxidant or anti-inflammation molecules include plant extracts like phenols, glycosides, aldehydes, alcohols, acids and other active substances [17] and micronutrients like selenium and vitamin E, as well as some probiotics with beneficial effects [16]. Malic acid (MA), an intermediate organic acid in the tricarboxylic acid cycle, exists widely in fruits and vegetables and can be mass-produced through microbial fermentation and enzymatic synthesis [18]. MA possesses excellent antioxidant and immune adjustment properties [19], and promoting-growth effects [20]. It has previously been demonstrated that MA could improve antioxidant capacity and enhance muscle fiber indices in weaned piglets and finishing pigs [21,22]. More recently, we found that maternal MA could have anti-inflammation and antioxidant effects on sows and their offspring and improve metabolic health in offspring, as well as improve reproductive performances in sows [23]. The modulation of the gut microbiota of the offspring through the vertical transmission of milk metabolites underlies the positive effects of MA on the offspring [23]. However, the mechanism behind the positive effects of MA in sows during late pregnancy has not been elucidated. We hypothesized that MA might ameliorate oxidative stress and inflammation in sows through modulating gut microbiota and host metabolic profiles during late pregnancy. Therefore, this study was to investigate the mechanism underlying the effects of maternal supplementation with MA during late pregnancy on oxidative stress and inflammation from the perspectives of maternal changes in the gut microbiota and serum metabolome.

## 2. Materials and Methods

### 2.1. Experimental Design and Diets

Animal experimentation protocols were kept strictly to the terms of the contract of the Care and Use of Laboratory Animals established by China Agricultural University (Protocol ID: SKLAB-2011-04-03) and the Animal Welfare and Ethical Committee of Beijing Academy of Agriculture and Forestry Sciences (approval number: IHVM11-2202-2). The experiment involved 20 multiparous sows (about 3.5 years old) with similar parities, backfat thickness, and expected farrowing dates. These sows were randomly allocated into two groups, each with ten replicates (*n* = 10), where each replicate consisted of one sow housed individually in a pen. The sows were provided with a basal diet (control group) or a basal diet supplemented with 2% L-malic acid complex during late pregnancy (from 85 days of gestation to parturition). The basal diet met the nutritional requirements recommended by the National Research Council for pregnant sows (NRC, 2012). The ingredients’ components and the nutrition level of the basal diet were shown as presented [23]. L-malic acid complex, containing 20% MA and 80% carrier (zeolite powder), was provided by Anhui Sealong Biotechnology Co., Ltd. (Bengbu, Anhui Province, China). During the experiment, each sow had ad libitum access to water and was fed 3 kg of diet daily (divided into two equal meals at 8:00 a.m. and 4:00 p.m., respectively). On day 107 of pregnancy, the sows were transferred to the farrowing rooms, where each sow was provided with an individual farrowing crate (2.1×1.5 m) and remained there until the weaning of the piglets.

### 2.2. Sample Collection

Blood sample at parturition was collected from ear veins and serum was obtained as previously [23]. Fresh fecal samples at parturition were collected and stored in sterile 2-mL centrifuge tubes at −80 °C for gut microbiota analysis.

### 2.3. DNA Extraction, 16S rRNA Sequencing of Gut Microbiota, and Data Analysis

Six fecal samples were randomly selected from each group. Total genomic DNA from fecal microbiota was extracted using a DNA extraction kit (M5636, Omega, Norcross, GA, USA). DNA concentration was measured using a spectrophotometer. DNA purity and quality were assessed by agarose gel electrophoresis.

PCR amplification was performed using universal primers targeting the V3 and V4 regions of bacterial 16S sequences. The upstream primer used was 338F (5′-ACTCCTACGGGAGGCAGCAG-3′), and the downstream primer was 338R (5′-GGACTACHVGGGTWTCTAAT-3′), resulting in amplicons of approximately 500 bp. PCR products were extracted from a 2% agarose gel and purified using the AxyPrep DNA Gel Extraction Kit (AP-GX-50, Axygen Biosciences, Union City, CA, USA). Purified amplicons were pooled in equal molars according to the standard protocol of Majorbio Bio-pharm Technology Co., Ltd. (Shanghai, China) and subjected to paired-end sequencing on the Illumina MiSeq PE300/NovaSeq PE250 platform (Illumina, San Diego, CA, USA).

The raw sequences were processed using the DADA2 plugin of QIIME2 software (https://qiime2.org/, accessed on 23 February 2023), including filtering, de-noising, merging, and non-chimeric removal processes. At a similarity level of 97%, the sequences were clustered into operational taxonomic units (OTUs). Representative OTU sequences were aligned against the Silva Release 138 database to obtain taxonomic annotations. Alpha diversity indices, including Shannon and Simpson indices, the Chao1 richness estimator, and abundance-based coverage estimator (ACE), were calculated using QIIME (version 1.9.1). Linear discriminant analysis (LDA) effect size (LEfSe, http://huttenhower.sph.harvard.edu/LEfSe, accessed on 23 February 2023) was performed to determine significant differences in bacterial taxa (from phylum to genus) between groups (LDA score > 2, *p* < 0.05).

### 2.4. Untargeted Metabolomics Analysis of Sows’ Serum

The relative concentration of metabolites in serum was quantified using the UHPLC-MS/MS system in both positive and negative ion modes. Briefly, a total of 400 μL of extraction solution (acetonitrile: methanol, 1:1, *v*/*v*) containing 0.02 mg/mL internal standard (L-2-chlorophenylalanine) was added to 100 μL of serum. After thorough mixing, the samples underwent a 30 min low-temperature ultrasonic extraction. After that, samples were kept at −20 °C for 30 min. Then, they were centrifuged at 13,000× *g* for 15 min at 4 °C. The supernatant was discarded, and the residue was dried with nitrogen gas. Subsequently, 100 μL of resuspension solution (acetonitrile:water, 1:1, *v*:*v*) was added. The samples underwent a second round of low-temperature ultrasonication, followed by centrifugation. The resulting supernatant was transferred to sample vials for subsequent analysis. The metabolites underwent separation on an HSS T3 column (100 × 2.1 mm; i.d., 1.8 μm) and were detected using mass spectrometry in both positive and negative ion scanning modes. An equal volume of all samples was mixed to prepare a quality control (QC) sample. During the analysis process, a QC sample was inserted every 5 samples to test the repeatability of the entire analysis process. The obtained raw data were then imported into the Progenesis QI software (Waters Corporation, Milford, MA, USA) for data preprocessing and annotated using HMDB (http://www.hmdb.ca/, accessed on 23 February 2023), Metlin (https://metlin.scripps.edu/, accessed on 23 February 2023), and Majorbio databases, as described previously [24]. Then, the data were analyzed using principal component analysis (PCA), partial least squares discriminant analysis (PLS-DA), or orthogonal partial least squares discriminant analysis (OPLS-DA). Metabolites exhibiting Variable Importance in Projection (VIP) > 1 and *p* < 0.05 were identified as significantly different. Differential metabolites were annotated and enriched using the KEGG database for metabolic pathway analysis.

### 2.5. Fecal Short-Chain Fatty Acid (SCFA) Analysis

HPLC-grade SCFA standards (>99% pure), including acetic acid, propionic acid, butyric acid, isobutyric acid, valeric acid, isovaleric acid, hexanoic acid, isohexanoic acid, and 1,3-butanediol (the internal standard), were purchased from Sigma-Aldrich (St. Louis, MO, USA). The SCFAs in feces were determined using a gas chromatograph with some modification. Briefly, 0.5 g of feces was placed in a 10 mL centrifuge tube and mixed with 10 mL of ultrapure water. After homogenization and centrifugation, the fecal supernatant was diluted with ultrapure water and filtered through a 0.22 μm filter. Filtrates were placed in headspace vials and injected into the Agilent 8890B gas chromatography system coupled to an Agilent 5977B/7000D mass selective detector with an inert electron impact (EI) ionization source at Majorbio Bio-Pharm Technology Co., Ltd. (Shanghai, China). The Dionex ICS-3000 ion chromatography system with an auto-sampler was used. The column used was the HP-FFAP (30 m × 0.25 mm × 0.25 µm) capillary column. The injector was split at 1:10. The carrier gas was nitrogen at a flow rate of 1.0 mL/min. Peaks corresponding to each SCFA were quantified using standard curves.

### 2.6. Statistical Analysis

Results were presented as means ± SEM and analyzed using the unpaired two-tailed Student’s *t*-test in SAS (v.9.1, SAS Institute, Cary, NC, USA). Relationships among key parameters were evaluated using Spearman’s correlation analysis. Procrustes function analysis within the R vegan package was used for Procrustes analysis of microbiome and metabolome data. Statistical significance was set at *p* < 0.05, while 0.05 ≤ *p* ≤ 0.10 was considered suggestive of a trend.

## 3. Results

### 3.1. Maternal MA Supplementation Modulated the Composition but Not the Richness or Diversity of Gut Microbiota during Late Pregnancy

**Figure 1:**
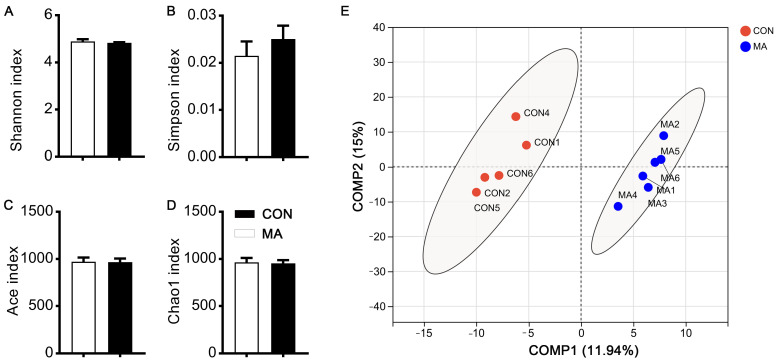
Influence of L–malic acid on gut microbiota diversity in sows during late pregnancy. (**A**–**D**) The α–diversity and (**E**) β–diversity of the gut microbiota in sows by 16S rRNA sequencing. (**A**–**D**) Comparison of Shannon index (**A**), Simpson index (**B**), Ace index (**C**), and Chao 1 index (**D**) between CON and MA groups. (**E**) Unweighted principal coordinate analysis (PCoA) at the OTU level. CON, basal diet; MA, basal diet containing 2% malic acid complex. Data were shown as means ± SEM (*n* = 6).

**Figure 2:**
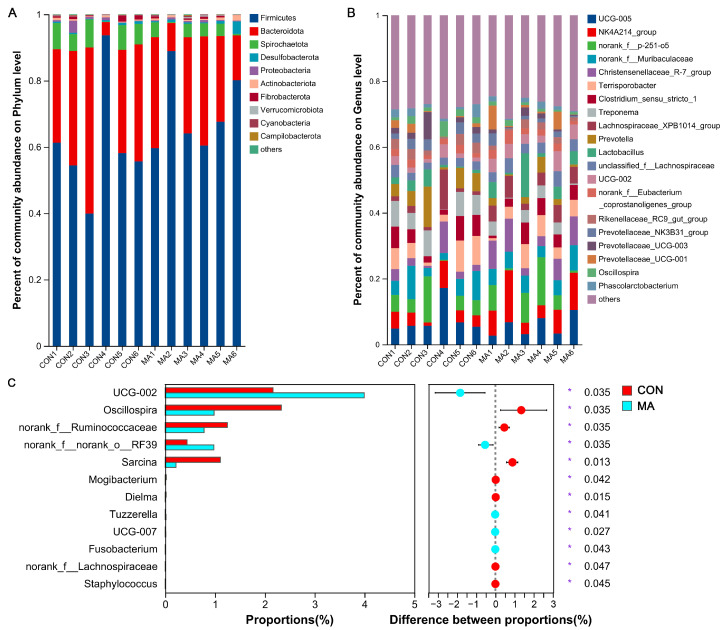
Modulation of L–malic acid on the constitution of gut microbiota in sows during late pregnancy by 16S rRNA sequencing: (**A**,**B**) shows the relative abundance of the bacteria community at the phylum and genus level, respectively; (**B**) displays the top 20 abundant genera; and (**C**) shows the communities with significant differences in relative abundance at genus level. CON, basal diet; MA, basal diet containing 2% L–malic acid complex. Data are presented as mean ± SEM (*n* = 6). Statistics were performed with Student’s *t*-test. * *p* < 0.05.

Previously, we showed that MA supplementation during late pregnancy improved antioxidant and inflammation status, and reproductive performances [23]; here, we tried to dissect the mechanism behind the MA’s positive effects on sows. Fecal microbiota from control and MA-treated sows were evaluated. Results showed that the α–diversity was not significantly changed, as indicated by the unaltered Shannon, Simpson, ACE, and Chao1 indices (Figure 1A–D), suggesting that maternal MA supplementation had no significant impact on gut microbiota species richness and diversity. Next, β–diversity was evaluated by unweighted principal coordinate analysis (PCoA) at the OTU level. A discernible separation of the MA group from the control group indicated that MA significantly modulated the composition of the gut microbiota (Figure 1E). At the phylum level, Firmicutes, Bacteroidota, and Spirochaetota were the top abundant phyla identified in late pregnancy for sows (Figure 2A). Among them, the abundance of Spirochaetota was significantly decreased with MA intervention. At the genus level, *UCG-005*, *NK4A214_group*, *norank_f__p-251-o5*, *norank_f__Muribaculaceae*, *Christensenellaceae_R-7_group*, *Terrisporobacter*, *Clostridium_sensu_stricto_1*, *Treponema*, *Lachnospiraceae_XPB1014_group*, *Prevotella*, and *Lactobacillus* were the top abundant microorganisms identified in late pregnancy for sows (Figure 2B). Specifically, the abundances of *Oscillospira*, *Ruminococcaceae*, and *Sarcina* were lower but *UCG-002* and *norank_o_RF39* were higher in the MA group compared to the control group (Figure 2C). There was also some microbiota with a significant difference in the two groups, such as *Mogibacterium*, *Dielma*, *Tuzzerella*, *UCG-007*, *Fusobaterium*, *Lachnospiraceae*, and *Staphylococcus*, although they did not occupy a high proportion (Figure 2C). Therefore, the aforementioned data suggest that dietary supplementation with MA during late pregnancy exerts a certain influence on the microbiota composition of sows.

### 3.2. Gut Microbiota Alteration May Mechanically Contribute to the MA-Enhanced Antioxidant and Anti-Inflammation Capacity

**Figure 3:**
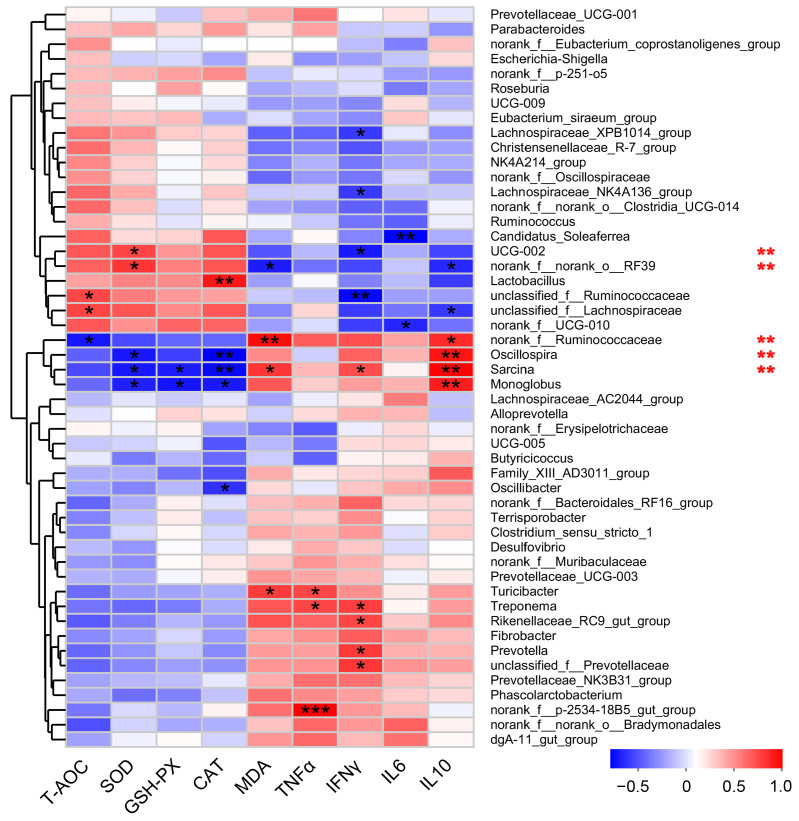
Correlation between top 50 abundant gut microbiota and antioxidant ability and inflammatory factors in the sows based on Spearman coefficient. Red indicates a positive correlation, and blue indicates a negative correlation. The star symbol in the small square presents the correlation results. * *p* < 0.05, ** *p* < 0.01, *** *p* < 0.001; ** on the right of the picture represents significantly different microorganisms between control and malic acid group.

Given that the gut microbiota plays a crucial role in the antioxidant and anti-inflammatory capabilities of sows and reproduction modulation, to assess the significance of the modulated gut microbiota underlying the MA’s positive effects, Spearman analysis between levels of the top 50 abundant microbes and our previously reported antioxidant enzymes and inflammatory factors [23] in the serum were analyzed (Figure 3). From the antioxidant perspective, the results revealed that the activity of the total antioxidant enzyme (T-AOC) was negatively correlated with the abundance of *norank_f_Ruminococcaceae* and positively correlated with *unclassified_f_Ruminococcaceae* and *unclassified_f_Lachnospiraceae*. The activity of superoxide dismutase (SOD) was positively correlated with *UCG-002* and *norank_f_norank_o_RF39* but negatively correlated with *Oscillospira*, *Sarcina*, and *Monoglobus*. Glutathione peroxidase (GSH-PX) was negatively linked to *Sarcina* and *Monoglobus*. Catalase (CAT) activity exhibited a negative correlation with *Oscillospira*, *Sarcina*, and *Monoglobus*, and a positive relationship with *Lactobacillus*. Malondialdehyde (MDA) content was positively correlated with *norank_f_Ruminococcaceae*, *Sarcina*, and *Turicibacter*. These data implied that *unclassified_f_Ruminococcaceae*, *unclassified_f_Lachnospiraceae*, *UCG-002*, *norank_f_norank_o_RF39*, and *Lactobacillus* play roles in antioxidation, while *norank_f_Ruminococcaceae*, *Oscillospira*, *Sarcina*, *Monoglobus*, and *Turicibacter* may promote oxidative stress. From the anti-inflammation perspective, we analyzed the correlations between levels of microbiota genera and anti-inflammation-related factors. The results indicated that TNF-α was positively related to *Turicibacter*, *Treponema*, and *norank_f__p-2534-18B5_gut_group.* IFN-γ exhibited a negative correlation with *Lachnospiraceae_XPB1014_group*, *Lachnospiraceae_NK4A136_group*, *UCG-002*, and *unclassified_f_Ruminococcaceae*, but a positive correlation with *Sarcina*, *Treponema*, *Rikenellaceae_RC9_gut_group*, *Prevotella*, and *unclassified_f_Prevotellaceae*. The IL6 level was negative with *Candidatus_Soleaferrea* and *norank_f_UCG-010*. IL-10 levels had positive correlations with the *norank_f_Ruminococcaceae*, *Oscillospira*, *Sarcina*, and *Monoglobus*, while had a negative correlation with the abundance of *norank_f_norank_o_RF39* and *unclassified_f_Lachnospiraceae*. These data implied that *Turicibacter*, *Treponema*, *norank_f__p-2534-18B5_gut_group*, *Sarcina*, *Treponema*, *Rikenellaceae_RC9_gut_group*, *Prevotella*, *unclassified_f_Prevotellaceae*, *norank_f_Ruminococcaceae*, *Oscillospira*, *Sarcina*, and *Monoglobus* are involved in inflammation, while *Lachnospiraceae_XPB1014_group*, *Lachnospiraceae_NK4A136_group*, *UCG-002*, *unclassified_f_Ruminococcaceae*, *Candidatus_Soleaferrea*, *norank_f_UCG-010*, *norank_f_norank_o_RF39*, and *unclassified_f_Lachnospiraceae* play an important role in the anti-inflammatory effect. Collectively, these data demonstrated that MA-induced gut microbiota alteration may mechanically contribute to the MA-enhanced antioxidant and anti-inflammation capacity.

### 3.3. MA Reshaped the Functions of Gut Microbiota

**Figure 4:**
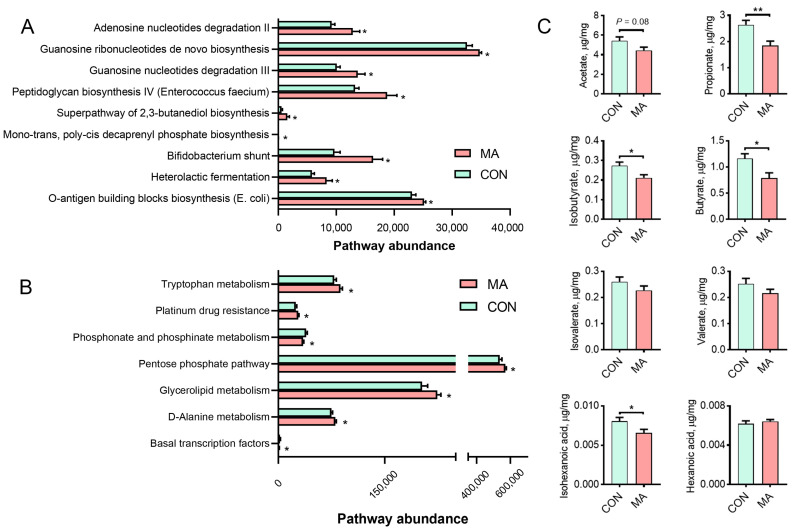
Functional prediction of fecal microbiota and the impact of L-malic acid on short-chain fatty acid content in feces. (**A**) Functional prediction of differential microbiota based on the MetaCyc database. (**B**) Differential functional analysis of metabolic pathways based on the KEGG Orthology database. (**C**) Levels of acetic acid, propionic acid, butyric acid, isobutyric acid, valeric acid, isovaleric acid, hexanoic acid, and isohexanoic acid in feces in the CON and MA groups. CON, basal diet; MA, basal diet containing 2% L-malic acid complex. Data are presented as mean ± SEM (*n* = 6). Statistics were performed with Student’s *t*-test. * *p* < 0.05, ** *p* < 0.01.

To investigate how MA-modulated gut microbiota work, we predicted the functional differences in gut microbiota using the PICRUST2 analysis. Analysis based on the MetaCyc revealed that fermentation and biosynthesis processes were enhanced with maternal MA supply during late pregnancy. The main biological pathways included O-antigen building block biosynthesis (*E*. *coli*), mono-trans, poly-cis decaprenyl phosphate biosynthesis, the super-pathway of 2,3-butanediol biosynthesis, peptidoglycan biosynthesis IV (*Enterococcus faecium*), and guanosine ribonucleotides de novo biosynthesis (Figure 4A). To further elucidate the roles of differential microorganisms, the enrichment of functional pathways was performed based on the KEGG Orthology database. Results showed that MA-regulated microorganisms mainly participated in the amino acid, lipid, and carbohydrate metabolic pathways, including D-alanine metabolism, glycerolipid metabolism, pentose phosphate pathway, phosphonate and phosphinate metabolism, platinum drug resistance, and tryptophan metabolism pathways (Figure 4B). Above all, these data suggested that maternal MA supply reshaped gut microbiota functions, by impacting their fermentation and biosynthesis functions, and improved substance metabolism.

### 3.4. MA Diminished SCFAs in Feces

One way in which the microbes work is through their metabolites, and one of their functions is to ferment carbohydrates to supply SCFAs for the host. Based on the facts that the fermentation and metabolic-related pathways of gut microbiota were influenced by MA, we next analyzed the SCFAS levels in feces. Results showed that, compared to the control group, the MA group exhibited a significant reduction in the levels of propionic acid, isobutyric acid, butyric acid, and isovaleric acid in feces. Acetic acid showed a decreasing trend (*p* = 0.08), while isocaproic acid, valeric acid, and caproic acid did not differ between the two groups.

### 3.5. MA Mediated the Metabolic Profile in Serum

**Figure 5:**
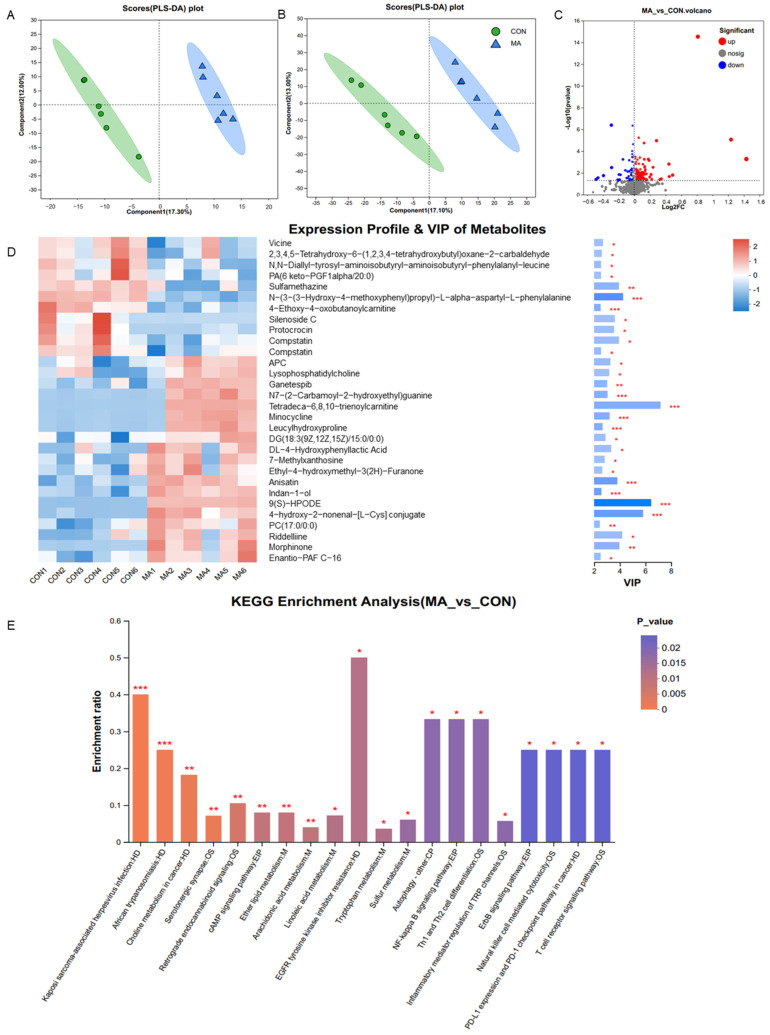
Effects of L-malic acid supplementation on the serum metabolic profiles of sows during late pregnancy. (**A**,**B**) PLS-DA plots of the metabolites in serum in positive ion and negative ion modes. (**C**) Volcano plots of the identified metabolites. The red ones represent upregulated metabolites and green ones represent downregulated metabolites. (**D**) VIP scores and the relevant abundance of the top 30 metabolites. The length of the bar represents the contribution of the metabolite to the difference between the two groups. * *p* < 0.05, ** *p* < 0.01, *** *p* < 0.001. (**E**) Enrichment of KEGG pathways based on the differential metabolites between the CON and MA groups. CON, basal diet; MA, basal diet containing 2% L-malic acid complex. OS, HD, M, EIP, and CP were the first categories of the KEGG pathway; they represent Organismal Systems, Human Diseases, Metabolism, Environmental Information Processing, and Cellular Processes, respectively.

Gut microbes influence host metabolism; given that gut microbiota was modulated by the MA supplementation, we further evaluated the changes in serum metabolic profiles using untargeted LC/MS analysis. A total of 1801 serum compounds were detected from the positive ion mode (809) and negative ion mode (992). The PLS-DA analysis was conducted to visualize the differences in all the samples among the two groups. The results showed a significant separation between the two groups (Figure 5A,B). In total, 155 metabolites were identified as differentially changed, including 116 upregulated and 39 downregulated (Figure 5C). They were annotated as presented in Supplemental Table S1. They mainly belonged to the groups of lipids and lipid-like molecules, organic acids and derivatives, organoheterocyclic compounds, benzenoids, and organic oxygen compounds. The top 30 metabolites responsible for the separation between groups were presented using their relative abundance and VIP scores (Figure 5D). The relative concentrations of vicine, 2,3,4,5-tetrahydroxy-6-(1,2,3,4-tetrahydroxybutyl)-oxane-2-carbaldehyde, N,N-Diallyl-tyrosyl-aminoisobutyryl-aminoisobutyryl-phenylalanyl-leucine, PA (6 keto-PGF1alpha/20:0), sulfamethazine, N-(3-(3-Hydroxy-4-methoxyphenyl)propyl)-L-alpha-aspartyl-L-phenylalanine, 4-ethoxy-4-oxobutanoylcarnitine, silenoside C, Protocrocin, compstatin, and Oc-tanoylcarnitine were decreased in the MA group compared to the CON group. Apc, lyso-phosphatidylcholine, ganetespib, N7-(2-Carbamoyl-2-hydroxyethyl)-guanine, tetradeca-6,8,10-trienoylcarnitine, minocycline, leucylhydroxyproline, DG (18:3(9Z,12Z,15Z)/15:0/0:0), DL-4-hydroxyphenyllactic acid, 7-methylxanthosine, ethyl-4-hydroxymethyl-3(2H)-furanone, anisatin, indan-1-ol, 9(S)-hpode, 4-hydroxy-2-nonenal-[L-Cys] conjugate, PC(17:0/0:0), riddelliine, morphinone, and Enantio-PAF C-16 were elevated by MA treatment. Pathway enrichment based on the KEGG database revealed that they are mainly involved in human diseases, genetic information processing, organismal systems, metabolism, and environmental information processing. Among them, metabolic pathways were mostly enriched, including amino acid, lipids, and the pyrimidine metabolism (Figure 5E). Interestingly, lysine degradation, linoleic acid metabolism, tryptophan metabolism, alanine, aspartate and glutamate metabolism, arginine biosynthesis, histidine metabolism, arginine and proline metabolism, choline metabolism in cancer, and serotonergic synapse were enriched. They accounted for a large proportion of the enriched metabolic pathways, highlighting the significance of amino acids behind the beneficial effects of MA during late pregnancy. In addition, arachidonic acid metabolism, a lipid metabolic pathway that functions in reproduction and metabolic health, was also enriched.

### 3.6. Correlation between Serum Metabolites and Fecal Microbiota/Antioxidant or Inflammation Indices

**Figure 6:**
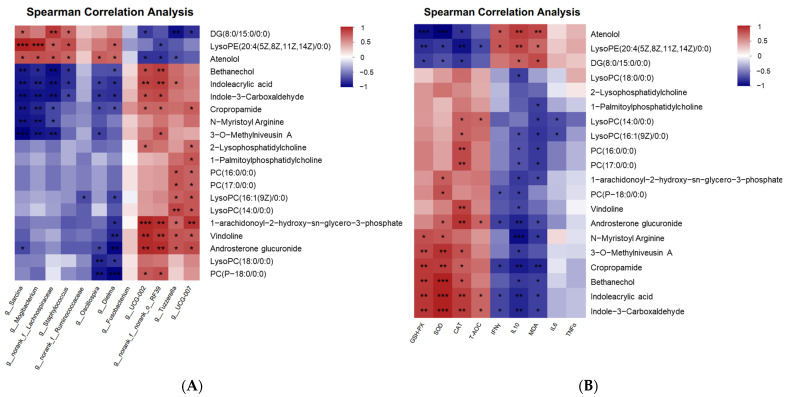
Correlation analysis between the top 20 abundant metabolites and differential microorganisms (**A**), and between the top 20 differential metabolite and antioxidant or inflammation indices (**B**) based on Spearman correlation coefficients. Red indicates a positive correlation, and blue indicates a negative correlation. * *p* < 0.05, ** *p* < 0.01, *** *p* < 0.001.

The links between the abundance of the top 20 abundant serum metabolites with the 12 different fecal microbes (at the genus level, *p* value < 0.05) and the antioxidant or inflammation indices were evaluated using Spearman’s correlation analysis. It is evident that there was a strong correlation between differential fecal microbiota and differential serum metabolites, as well as between serum metabolites and antioxidant (or inflammation) indices. As shown in Figure 6A, on the one hand, the positive correlation was between microbiota *saecina*, *Mogibacterium*, *Lachnospiraceae*, *Staphylococcus*, *Ruminococcaceae*, *Oscillospira*, *Dielma*, and metabolites DG (8:0/15:0/0:0), LysoPE (20:4(5Z,8Z,11Z,14Z)/0:0), and atenolol. And the other five microbes, *Fusobacterium*, *UCG-002*, *RF39*, *Tuzzerella*, and *UCG-007*, were positive with the majority of different metabolites, such as 1-arachidonoyl-2-hydroxy-sn-glycero-3-phosphate, vindoline, and androsterone glucuronide. On the other hand, the relative abundances of *saecina*, *Mogibacterium*, *Lachnospiraceae*, *Staphylococcus*, *Ruminococcaceae*, *Oscillospira*, and *Dielma* were negatively correlated with the concentration of the above majority of different metabolites. And the metabolites DG (8:0/15:0/0:0), LysoPE (20:4(5Z,8Z,11Z,14Z)/0:0), and atenolol, and microbiota *Fusobacterium*, *UCG-002*, *RF39*, *Tuzzerella*, and *UCG-007*, were negatively correlated. On the other hand, these differential metabolites with higher abundance have a close relationship with anti-oxidative enzymes and inflammatory factors. To be specific, the activity of GSH-PX, SOD, CAT, and T-AOC were negatively related to atenolol, LysoPE (20:4(5Z,8Z,11Z,14Z)/0:0), and DG (8:0/15:0/0:0). And the levels of IFN-γ, IL10, and MDA were positively related to atenolol, LysoPE (20:4 (5Z,8Z,11Z,14Z)/0:0), and DG (8:0/15:0/0:0). Furthermore, indole-3-carboxaldehyde, indoleacrylic acid, bethanechol, cropropamide, 3-O-methylniveusin A, N-myristoyl arginine, androsterone glucuronide, vindoline, PC(P-18:0/0:0), 1-arachidonoyl-2-hydroxy-sn-glycero-3-phosphate, PC (17:0/0:0), PC (16:0/0:0), LysoPC (16:1(9Z)/0:0), LysoPC (14:0/0:0), 1-Palmitoylphosphatidylcholine, and LysoPC (18:0/0:0) might have anti-oxidative and anti-inflammatory properties because they were positively connected with anti-oxidative enzymes and negative correlated to pro-inflammatory factor. Collectively, the changes in serum metabolites are closely associated with alterations in gut microbiota. Furthermore, the variations in metabolites are linked to enhancements in host antioxidant and anti-inflammatory capabilities.

## 4. Discussion

During late pregnancy, the fetus undergoes rapid growth and the metabolic intensity of the mother’s body increases [25,26]. Consequently, the mother may experience low-level inflammation and significant oxidative stress, which adversely affect pregnancy outcomes [27,28]. Due to the specificity and sensitivity of the pregnancy stage, the exploration of safe and healthy nutritional antioxidants is crucial for mitigating the adverse consequences of various stress issues in late pregnancy. Our preliminary findings have revealed that the increased litter size in sows with MA intervention is attributed to the enhancement of the sow’s anti-inflammatory and antioxidant capabilities [23] because supplementing the maternal diet with MA during the late gestation period significantly enhances the antioxidant enzyme and decreases pro-inflammatory factors in the serum of sows at farrowing [23]. In this study, we further elucidate the mechanism of MA’s anti-inflammatory and antioxidant effects from the perspectives of microbial metabolism and maternal metabolism.

The intestinal microbiota undergoes changes during pregnancy to adapt to the evolving needs of both the mother and fetal development [29]. We observed that, although the intervention of MA in the late stages of pregnancy did not alter the richness and diversity of the microbial community, it did reshape the composition of the intestinal microbiota, leading to significant changes in the abundance of specific bacterial species. We found that Firmicutes, Bacteroidota, and Spirochaetota were the top abundant phyla in late pregnancy for sows. Consistent with our study, Shao et al. reported that Firmicutes, Bacteroidetes, Proteobacteria, and Spirochaetes were the top four predominant flora [8]. Zhou et al. reported that Firmicutes, Bacteroidetes, and Spirochaetes were the top three predominant flora [30], and Kong et al. reported that Firmicutes and Bacteroidetes were the top two predominant flora in pregnant sows. More specifically, the relative abundance of Firmicutes accounted for over 50%, and Bacteroidetes, Proteobacteria, and Spirochactes followed [8]. Analogously, Firmicutes, Bacteroidetes, and Euryarchaeota were found to be the top three abundant phyla in pregnant cows, accounting for 48.68%, 34.45%, and 15.42% of the total microbiota, respectively [29]. Although there was some inconformity for the Treponema, Clostridium, Ruminococcus, Proteobacteria, and Euryarchaeota, these data suggest that Firmicutes and Bacteroidetes are the abundant phyla for the pregnant sows. It has been reported that the dominant taxa belonging to Firmicutes in the digestion are associated with the distal colon and likely exhibit an important function in starch and fiber degradation [31]. Bacteroidetes can degrade indigestible dietary polysaccharides into short-chain fatty acids [32]. Their abundance is associated with the energy metabolism of the host [33]. Therefore, these findings indicate that host–microbial interactions promote adaptations to pregnancy and will support the development of the metabolic changes to the fetus and the mother for energetic demands to achieve optimal pregnancy outcomes.

The intestinal microbiota is closely associated with oxidative stress and inflammation and many exogenous additives regulate oxidative stress and inflammation levels in the body by modulating the structure of the intestinal microbiota [34,35]. Dietary fiber composition in the gestational diet of sows can modulate the antioxidant capacity and inflammatory responses of both the mother and offspring by regulating the composition of the intestinal microbiota [36]. A study showed that reduced oxidative stress and inflammation are probably related to the adjusted *Ruminococcaceae UCG-008* and the *Christensensllaceae R-7 group* in sows supplemented with resveratrol [37]. Based on the correlation analysis between microbiota and antioxidants, our study suggests that *UCG-002*, *RF39*, *Lactobacillus*, *unclassified_f_Ruminococcaceae*, *norank_f_UCG-010*, *and unclassified_f_Lachnospiraceae* may play a role in antioxidant and anti-inflammation effects. It has been shown that *UCG-002* (family *Oscillospiraceae*) is typically associated with higher growth performance in growing pigs [38,39]. A low level of inflammatory response and a high level of antioxidant ability leads to higher performance. A study showed that a high-fiber diet can increase the abundance of *RF39*, which may play a role in inhibiting the overgrowth of *E.coli* and negatively relate to LPS biosynthesis [40]. *Lactobacilllus* is generally regarded as a safe and beneficial probiotic in animals [41]. *Ruminococcaceae* and *Lachnospiraceae* constitute the main families in the mammalian gut and link to the preservation of gut health [42]. Moreover, the abundance of these *UCG-002* and *RF39* was significantly higher in the MA group compared to the control group, suggesting that the enhancement of antioxidant and anti-inflammatory capacity may be caused by *UCG-002* and *RF39* in pregnant sows supplemented with MA. The genera *norank_f_Ruminococcaceae*, *Oscillospira*, *Sarcina*, *Monoglobus*, *Osillibacter*, and *Turicibacter* may contribute to oxidative stress and inflammation. There are also some species of *Ruminococcaceae* related to lipopolysaccharide (LPS) biosynthesis [43]. Furthermore, the genus *Sarcina* (Family *Clostridiaceae*) was a pathogen that related to gastric dilation and emphysematous gastritis in humans and animals [44]. *Treponema*, *Rikenellaceae_RC9_gut_group*, *Prevotella*, *unclissified_f_Prevotellaceae*, and *norank_f_p-2534-18B5_gut_group* also demonstrated significant positive correlations with pro-inflammatory factors, which may contribute to the inflammatory reaction in the CON group. *Treponema* was one of the main pathogens in *Spirochaetes*, and is related to colon inflammation [45,46]. The relative abundance of *Rikenellaceae_RC9_gut_group* will increase deeply after LPS exposure and its surge will exacerbate intestinal inflammation so that it will serve as a biomarker of intestinal damage [47]. Furthermore, the increase in the *Rikenellaceae_RC9_gut_group* is related to abnormal glucose and lipid metabolism [48]. Moreover, the relative abundances of *norank_f_Ruminococcaceae*, *Oscillospira*, and *Sarcina* were significantly higher in the control group than in the MA group, indicating that their increase might lead to inflammatory and oxidative stress in pregnant sows. In the broilers fed with bio-fermented MA, some bacteria such as *norank_f_norank_o_RF39*, *Tuzzerella*, and *Oscillospira* also show a significant change in the cecum [49]. These findings suggest the reshaped intestinal microbiota composition modulates the MA’s oxidative stress and inflammation modulation effects during late pregnancy in sows.

Through microbial functional prediction, we identified that MA induced microbiota-related functional alterations. Fermentation and biosynthesis functions of microbes and substance metabolism, such as amino acids, lipids, and carbohydrates metabolism, were enriched. Correspondingly, SCFAs, one series of metabolites of microorganisms fermented based on intestinal nutrients [50], were decreased in the MA group. To the best of my knowledge, this study is the first to investigate the impact of MA supplementation on fecal SCFAs. According to reports, microbes that produce SCFAs are mainly comprised of *Bacteroides*, *Bifidobacterium*, *Eubacteria*, *Streptococcus*, and *Lactobacillus* [51]. In our study, *Oscillospira*, one potent risk factor for constipation [52,53], which can produce SCFAs, such as butyrate and propionate [54], was decreased in the MA group, which may partially explain the decrease in SCFAs induced by MA. But it should be noted that SCFAs are commonly associated with better host redox status and inflammation levels [55]; therefore, this finding suggests that the beneficial effects of MA might be independent of the decreased SCFAs. In addition, methylamines generated from choline and indoles derived from dietary tryptophan metabolism were also important gut microbial metabolites to modulate host–microbe interactions [56,57]. As a tryptophan metabolite, indoleacrylic acid has been proven to have the function of suppressing inflammation and promoting gut health [58], and was significantly upregulated in the MA group. Another increased Trp metabolite in our study, indole-3-carboxaldehyde, is reported to activate aryl hydrocarbon receptor receptors and inhibit intestinal inflammation [59]. Collectively, this finding demonstrated that MA enhanced microbiota metabolism, contributing to the improved inflammation status.

The gut microbiome and host interact with each other. By deepening evidence of the mechanisms underlying the interactions between the microbiota and its host [60], we further explored the changes in the host’s metabolic profile after MA treatment. We identified differential metabolites between the two groups and revealed that the changes in the gut microbiome were correlated with the alterations in serum metabolites. We found that MA significantly reshaped the host metabolism, primarily involving the amino acid, lipid, and pyrimidine metabolisms. The two main pathways of metabolite enrichment were choline metabolism in cancer and glycerophospholipid metabolism. Glycerophospholipids, which mainly include PE, PS, PC, and others, are fundamental components of biofilm systems, and they play a role in transcription, energy metabolism, and signal transduction [61,62]. A study showed that dietary MA supplementation in tilapia can improve hepatic lipid accumulation [63]. These data suggest a high association between MA and host lipid metabolism. The enrichment of metabolites associated with lipid metabolism before farrowing in our study is likely advantageous for the host in terms of milk synthesis and lactation. This is attributed to the substantial energy requirements associated with milk production.

More interestingly, we found that amino acid metabolism plays an important role in the beneficial role of MA, as indicated by the fact that many amino-acid-metabolism-related pathways were enriched by MA treatment. Among them, tryptophan is one of the essential amino acids in animals, and research indicates that tryptophan and its metabolites are effective in scavenging free radicals, and functioning as robust antioxidants [64,65]. Tryptophan metabolism is closely linked to gut health, and tryptophan deficiency can promote the development of inflammatory bowel disease (IBD) [66]. Research indicates a significant increase in the biosynthesis processes of tryptophan, tyrosine, and phenylalanine as pregnancy progresses in sows [67]. This might be associated with promoting vascular development in sows, meeting the demands for the rapid growth of the fetus. In addition, histidine metabolism was also enriched in the MA group. As an essential amino acid, histidine was documented to improve antioxidant capacity in the liver through the endogenous synthesis of glutathione and NO [68]. Although a high level of histidine load (5 g/g BW) in pregnant rats could impair energy homeostasis in the cerebral cortex and hippocampus, and induce oxidative stress in the offspring [69], appropriate maternal histidine supply was related to enhanced antioxidant capacity and anti-inflammation status [70]. Of note, arginine and proline metabolism are importantly implicated in pregnancy and in protecting the host from damage [71]. They possess ROS scavenging properties, helping to maintain stable glutathione levels [72], and proline is inhibitory for liver inflammation [73]. In addition, we have observed that the changes in host metabolic products induced by MA are significantly correlated with alterations in gut microbiota as well as anti-inflammatory and antioxidant responses. Therefore, we proposed that the beneficial effects of MA were also related to metabolic modulation, especially lipid and amino acid metabolism.

## 5. Conclusions

We previously found that MA alleviated oxidative stress and inflammation, and improved reproductive performance in sows. However, the probable mechanism behind these positive effects remains unexplored. Here, we investigated the mechanism underlying the MA’s positive effects from the gut microbiota and host metabolic perspectives. Maternal MA supplementation modulated the composition but not the richness and diversity of gut microbiota during late pregnancy. Correlation analysis revealed a high correlation between gut microbiota and antioxidant capacity (or inflammation indicators). The metabolic landscape was modulated by MA and highly related to MA-induced changes in gut microbiota and antioxidant capacity (or inflammation indicators). Overall, our data demonstrated that a maternal dietary supply of MA could ameliorate oxidative stress and inflammation in sows, possibly through modulating gut microbiota and host metabolic profiles during late pregnancy.

## Data Availability

Data are contained within the article.

## Supplementary Materials

The following supporting information can be downloaded at: https://www.mdpi.com/article/10.3390/antiox13020253/s1, Table S1: Annotation information of the differential metabolites.
